# Ageritin—The Ribotoxin-like Protein from Poplar Mushroom (*Cyclocybe aegerita*) Sensitizes Primary Glioblastoma Cells to Conventional Temozolomide Chemotherapy

**DOI:** 10.3390/molecules27082385

**Published:** 2022-04-07

**Authors:** Rossella Rotondo, Sara Ragucci, Salvatore Castaldo, Nicola Landi, Maria Antonietta Oliva, Paolo V. Pedone, Antimo Di Maro, Antonietta Arcella

**Affiliations:** 1 IRCCS Istituto Neurologico Mediterraneo NEUROMED, Via Atinense 18, 86077 Pozzilli, Italy; rossellaross1988@gmail.com (R.R.); castaldosal90@gmail.com (S.C.); mariaantonietta.oliva@neuromed.it (M.A.O.); 2 Department of Environmental, Biological and Pharmaceutical Sciences and Technologies (DiSTABiF), University of Campania “Luigi Vanvitelli”, Via Vivaldi 43, 81100 Caserta, Italy; sara.ragucci@unicampania.it (S.R.); nicola.landi@unicampania.it (N.L.); paolovincenzo.pedone@unicampania.it (P.V.P.)

**Keywords:** ageritin, *Cyclocybe aegerita*, γ-H2AX, MGMT, micronuclei, patient-derived glioblastoma cell lines, ribotoxin-like proteins, temozolomide

## Abstract

Here, we propose Ageritin, the prototype of the ribotoxin-like protein family, as an adjuvant treatment to control the growth of NULU and ZAR, two primary human glioblastoma cell lines, which exhibit a pharmacoresistance phenotype. Ageritin is able to inhibit NULU and ZAR growth with an IC50 of 0.53 ± 0.29 µM and 0.42 ± 0.49 µM, respectively. In this study, Ageritin treatment highlighted a macroscopic genotoxic response through the formation of micronuclei, which represents the morphological manifestation of genomic chaos induced by this toxin. DNA damage was not associated with either the deregulation of DNA repair enzymes (i.e., ATM and DNA-PK), as demonstrated by quantitative PCR, or reactive oxygen species. Indeed, the pretreatment of the most responsive cell line ZAR with the ROS scavenger N-acetylcysteine (NAC) did not follow the reverse cytotoxic effect of Ageritin, suggesting that this protein is not involved in cellular oxidative stress. Vice versa, Ageritin pretreatment strongly enhanced the sensitivity to temozolomide (TMZ) and inhibited MGMT protein expression, restoring the sensitivity to temozolomide. Overall, Ageritin could be considered as a possible innovative glioblastoma treatment, directly damaging DNA and downregulating the MGMT DNA repair protein. Finally, we verified the proteolysis susceptibility of Ageritin using an in vitro digestion system, and considered the future perspective use of this toxin as a bioconjugate in biomedicine.

## 1. Introduction

Ribotoxin-like proteins (RL-Ps) are specific ribonucleases, isolated from fruiting bodies of edible basidiomycetes mushrooms. These enzymes damage ribosomes through the catalysis of the 23-28S rRNA endonucleolytic cleavage at a specific site within the Sarcin-Ricin Loop (SRL), causing the inhibition of translation and cell death [1]. The members of this family are non-glycosylated, basic (pI > 9.0), and monomeric proteins of ~135 amino acid residues with a single reactive free cysteinyl residue at the N-terminal region [2]. Additionally, some RL-Ps display metal-dependent endonuclease activity on plasmid DNA. The prototype of these enzymes is Ageritin, isolated from *Cyclocybe aegerita* [3], and structurally and functionally well characterized. In particular, Ageritin has a vacuolar localization in hyphae, is synthetized as a pre-form with a signal N-terminal peptide removed by proteolysis during post-translation [4,5], and is a very stable protein (Tm = 78 °C; Cm > 5.0 M) [6,7]. Moreover, the catalytic site consists of a catalytic triad made of one histidinyl (H75) and two aspartyl residues (D62 and D64) [7,8,9]. Several antipathogenic activities are attributed to Ageritin, such as antifungal, antibacterial, entomotoxic and nematotoxic activity [9,10,11], likely correlated to the defense machinery of *C. aegerita* mushroom, although the biological function of Ageritin is unknown.

Nowadays, considering the possible biotechnological application of these toxins, homologous members of RP-Ls that display structural features and cytotoxic action similar to Ageritin have recently been isolated from fruiting bodies of other edible mushrooms such as *Pleurotus ostreatus* [12], *Boletus edulis* [13] and *Calocybe gambosa* [2].

From a biomedical point of view, Ageritin and RP-Ls are of interest due to the in vitro antiproliferative activities towards different tumor cell lines [3,10]. In particular, the cytotoxic effect of Ageritin has been mainly evaluated against SH-SY5Y human neuroblastoma cells used as a model to study brain tumors, testing the effect on both undifferentiated and retinoic acid-differentiated forms [14]. Among brain tumors, glioblastoma multiforme (GBM) represents the most aggressive primary malignant tumor affecting the central nervous system [15]. Currently, GBM treatment includes surgery, radiotherapy, and alkylating chemotherapy with temozolomide (TMZ)—the latter has limited success in increasing overall survival. Given the poor prognosis of these tumors, there is a pressing need to look for new drugs and strategies to complement traditional therapy [16,17].

In this framework, we decided to analyze the effect of Ageritin on two primary lines, prepared from the biopsies of Neuromed patients (NULU and ZAR), whose heterogeneity reproduce the parental tumor from which they derived [18]. Furthermore, considering the mechanisms of glioblastoma primary cell line drug resistance to the alkylating agent TMZ [18], we verified the cytotoxic effect of Ageritin in the presence of TMZ on selected glioblastoma primary cell lines.

Finally, due to Ageritin’s cytotoxic effect, in light of its possible use alone or linked to antibodies (i.e., immunoconjugates) for GBM-targeted therapy, we evaluate its resistance to proteolysis in vitro.

## 2. Results

### 2.1. Susceptibility of Ageritin to Proteolysis

Considering the possible application of Ageritin as a biomedical tool, we verified its susceptibility to proteolysis [19].

**Figure 1:**
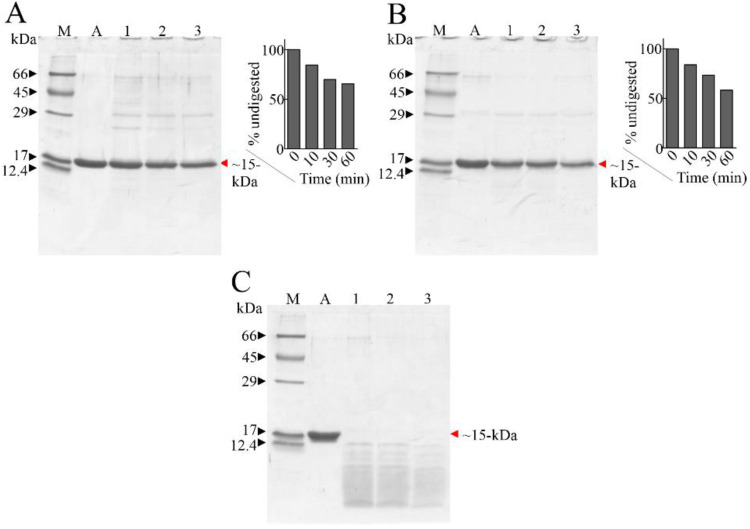
SDS-PAGE of Ageritin digested by proteases. Panel (**A**): 5 µg of Ageritin (A, negative control); lanes 1, 2 and 3, trypsin inhibited after 10 min, 30 min and 60 min, respectively. Panel (**B**): 5 µg of Ageritin (A, negative control); lanes 1, 2 and 3, chymotrypsin inhibited after 10 min, 30 min and 60 min, respectively. Insert of both panel a and b, densitometric analysis. Panel (**C**): 5 µg of Ageritin (A, negative control); lanes 1, 2 and 3, pepsin inhibited after 10 min, 30 min and 60 min, respectively. M, molecular weight markers; denatured SDS-PAGE was carried out in 15% polyacrylamide separating gel in reducing conditions (β-mercaptoethanol).

In this framework, the resistivity of Ageritin to proteases was investigated in vitro by using pepsin at acid pH as well as trypsin or chymotrypsin at neutral pH. The hydrolysis of Ageritin was ascertained by SDS-PAGE, as displayed in Figure 1. Data showed that when Ageritin was incubated with trypsin or chymotrypsin, it exhibited evident resistance (Figure 1A,B); however, when incubated with pepsin, it was susceptible to rapid degradation (Figure 1C). Therefore, in the presence of trypsin or chymotrypsin after 60 min, over 60% of the Ageritin band remained intact, as shown by the densitometric analysis (insert of Figure 1A,B). This resistivity to proteases at neutral pH is interesting, considering that trypsin and trypsin-like enzymes, as well as chymotrypsin and chymotrypsin-like proteases, are well characterized in the brain [20] and neuronal human tissues [21].

### 2.2. Cytotoxicity of Ageritin on Patient-Derived Glioblastoma Cell Lines NULU and ZAR

**Figure 2:**
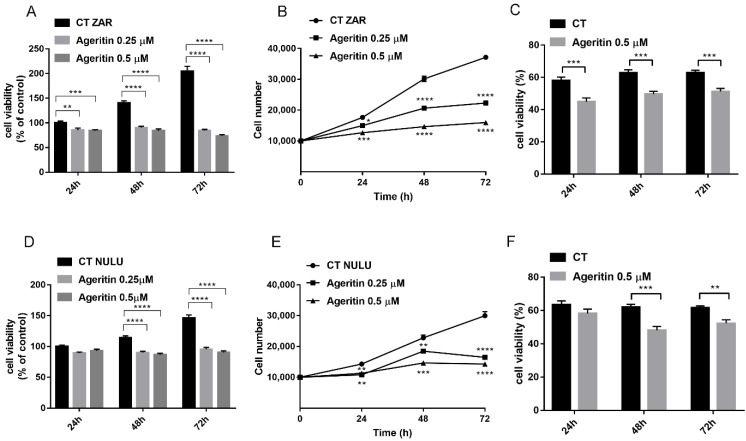
Dose-dependent cytotoxic effects of Ageritin on patient-derived glioblastoma cell lines. (**A**,**D**) MTT assay of ZAR and NULU cell lines daily treated with Ageritin 0.25 µM and 0.5 µM for 24, 48 and 72 h, respectively. Untreated cells were used as control. (**B**,**E**) growth curve of ZAR and NULU cell lines treated with Ageritin 0.25 µM and 0.5 µM, respectively. Untreated cells were used as control. (**C**,**F**) trypan blue assay reported as percentage of ZAR and NULU cell viability treated with Ageritin (0.5 µM) at 24, 48 and 72 h, respectively. For all the experiments, values are the means ± SEM of 3 individual determinations. Unpaired *t*-test, *p*-value < 0.05. According to GraphPad Prism 7, ** *p*-value 0.001 to 0.01 (very significant), *** *p*-value 0.0001 to 0.001 (extremely significant), **** *p*-value < 0.0001 (extremely significant).

According to a previous study in which Ageritin was tested towards commercial human U-251 glioma cells [3], we verified the cytotoxic effect of Ageritin on ZAR and NULU patient-derived glioblastoma cell lines, determining IC_50_-values at 48 h of 0.42 ± 0.49 µM and 0.53 ± 0.29 µM, respectively. Ageritin primary glioblastoma cell line treatment revealed the ability of this RL-P to suppress cell metabolism and inhibit cell growth in a dose-dependent manner. Indeed, cell treatment with Ageritin (0.25 and 0.5 µM) at 48 and 72 h dramatically affected the capacity of mitochondrial dehydrogenase enzymes to oxidize the tetrazolium to formazan salts (Figure 2A,D). In this regard, according to IC_50_-values, the responsiveness of ZAR cell line to Ageritin was higher than the NULU cell line, showing a ZAR cell line viability reduction of 49% and 65% at 48 and 72 h, respectively, in contrast with the NULU cell line residual viability of 24% and 38%, considering the higher concentration of RL-P (0.5 µM), with respect to control (Figure 2A,D).

Moreover, as evidenced in Figure 2B,E, growth curves determined by cell counting confirmed that ZAR was more sensitive than the NULU cell line to Ageritin treatment. Indeed, the higher concentration of RL-P (0.5 µM) reduced ZAR cell growth more than 50% (at 48 h and 72 h), while NULU cell growth showed a reduction around 40% (at 48 and 72 h). Finally, as shown by the trypan blue exclusion method, the percentage of dead cells constantly decreased at about 20% for both tested cell lines at 24, 48 and 72 h, considering the higher concentration of RL-P (0.5 µM) with respect to the control (Figure 2C,F).

### 2.3. Effect of Ageritin on Cell Migration and Invasion

**Figure 3:**
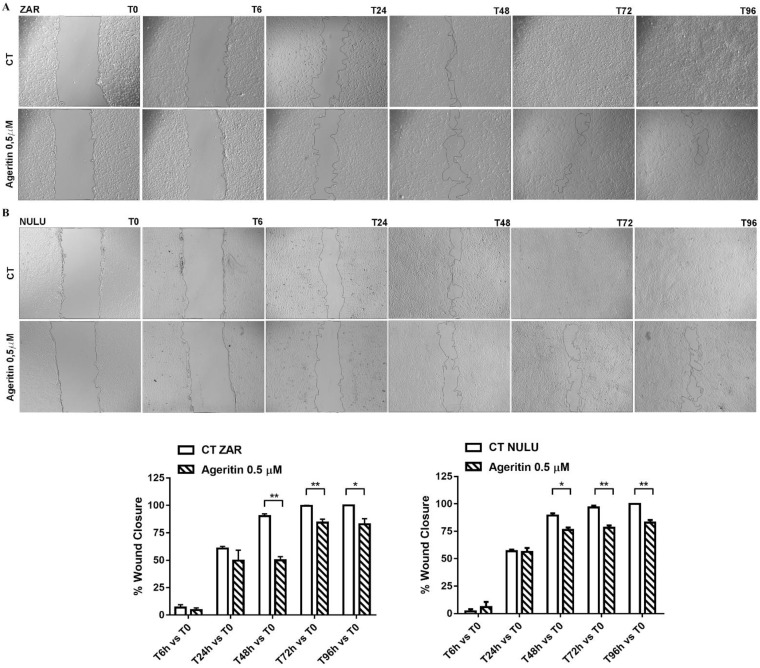
Wound healing assay on primary glioblastoma cell lines NULU and ZAR under treatment with Ageritin (0.5 μM). (**A**,**B**) Representative images of ZAR and NULU cell wound healing at 0, 6, 24, 48, 72 and 96 h, under Evos FL microscope (4× magnification). Graphs reported the quantification of the wound healing assays as percentage of wound closure. Data are reported as mean ± SEM of 3 individual determinations. Unpaired *t*-test, *p*-value < 0.05. According to GraphPad Prism 7, * *p*-value 0.01 to 0.05 (significant), ** *p*-value 0.001 to 0.01 (very significant).

A wound healing assay was used to determine the effect of Ageritin on the migration and invasion of ZAR and NULU patient-derived glioblastoma cell lines with respect to the control, as shown in Figure 3A,B, respectively. The percentage of wound closure significantly decreased when both tested cell lines were treated with 0.5 µM Ageritin, starting 48 h post-scratch. In detail, the percentage of wound closure in Ageritin-treated ZAR cell line with respect the control was about 49% vs. 90% at 48 h, and 84.3% vs. 99.5 % at 72 h, while the Ageritin-treated NULU cells versus control was about 76% vs. 89.4% at 48 h, and 78% vs. 96% at 72 h. For both cell lines, the scratch remained still open after 96 h (graphs in Figure 3A,B).

### 2.4. Ageritin-Induced Genotoxicity Evaluable as Increase in γ-H2AX (+) Micronuclei

**Figure 4:**
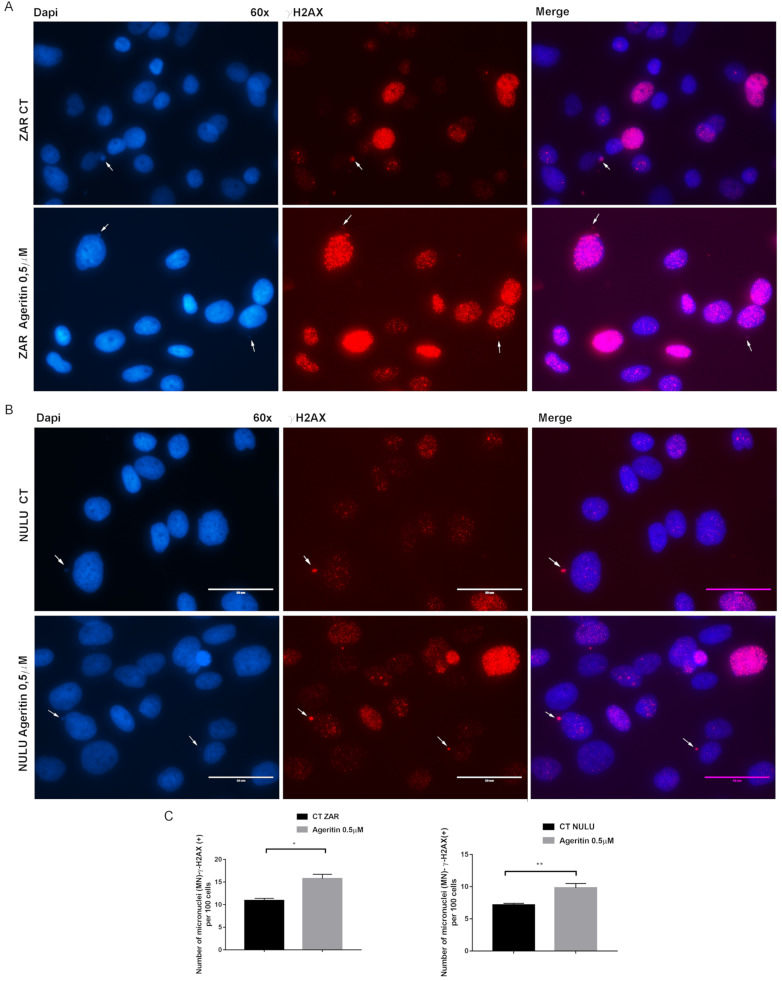
Representative immunofluorescence images of ZAR and NULU treated with Ageritin (0.5 μM) for 48 h. (**A**,**B**) Cell nuclei were stained with DAPI (left panels) and γ-H2AX (middle panels). White arrows indicate γ-H2AX (+) micronuclei. Merged images from representative cells were also shown (right panels). Magnification 60×. Scale bar represents 50 μm for all panels. (**C**) Quantification of the number of MN-γ-H2AX (+) per 100 mononucleated cells. Data are reported as mean ± SEM of 3 individual determinations. Unpaired *t*-test, *p*-value < 0.05. According to GraphPad Prism 7, * *p*-value 0.01 to 0.05 (significant), ** *p*-value 0.001 to 0.01 (very significant).

As a reliable biomarker of exposure to genotoxic agents, NULU and ZAR patient-derived glioblastoma cell lines, treated for 48 h with 0.5 µM Ageritin, were DAPI-screened to characterize micronuclei (MN) formation (Figure 4A,B, left panels). The preliminary evidence of Ageritin-induced genotoxicity was confirmed by γ-H2AX staining, a marker of DNA double-strand breaks (Figure 4A,B, middle panels). Merged images from representative cells were also shown (Figure 4A,B, right panels). Following the treatment with Ageritin, the frequency of MN-γ-H2AX (+) increased by 3.5-fold and 1.36-fold in ZAR and NULU cell lines, respectively, with respect to the untreated cells used as the control (Figure 4C).

**Figure 5:**
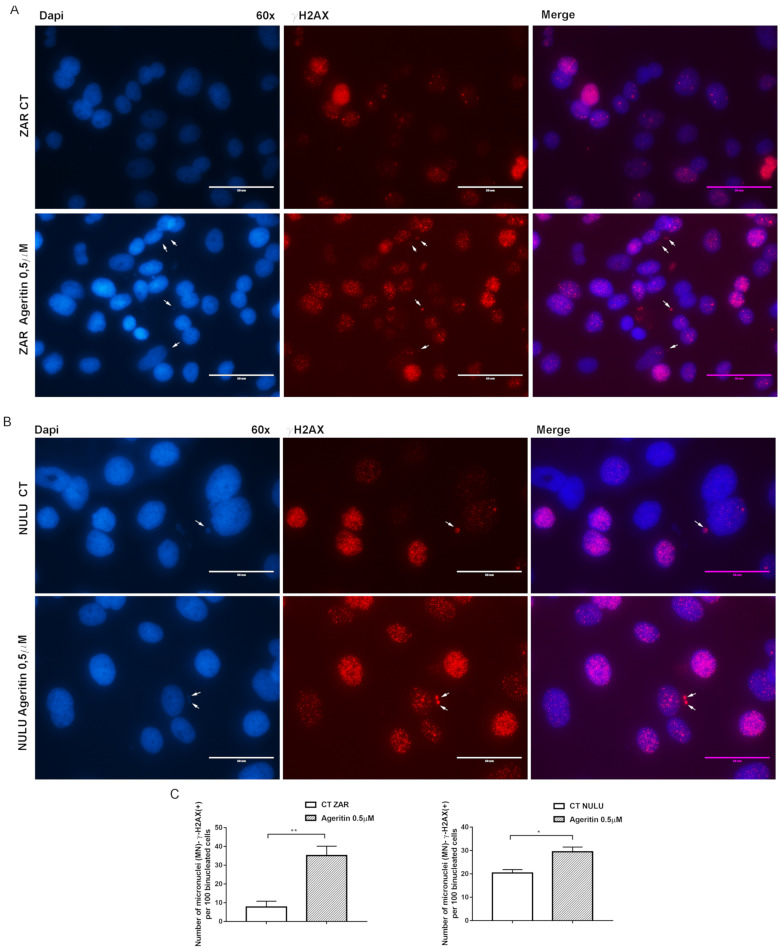
Representative immunofluorescence images of ZAR and NULU pre-treated with Cytochalasin B followed by Ageritin (0.5 μM) for 48 h. (**A**,**B**) Cell nuclei were stained with DAPI (left panels) and γ-H2AX (middle panels). White arrows indicate γ-H2AX (+) micronuclei. Merged images from representative cells were also shown (right panels). Magnification 60×. Scale bar represents 50 μm for all panels. (**C**) Quantification of the number of MN-γ-H2AX (+) per 100 binucleated cells. Data are reported as mean ± SEM of 3 individual determinations. Unpaired *t*-test, *p*-value < 0.05. According to GraphPad Prism 7, * *p*-value 0.01 to 0.05 (significant), ** *p*-value 0.001 to 0.01 (very significant).

To further confirm the genotoxic effects, micronuclei assay with Cytochalasin B was performed (Figure 5A,B). Data revealed that the frequency of MN-γ-H2AX (+) for 100 binucleated cells of both ZAR and NULU cell lines was comparable to that reported below for mononucleated cells (Figure 5C).

### 2.5. Transcriptional Deregulation Analysis of DNA Repair Enzyme Ataxia-Telangiectasia-Mutated ATM and DNA-Dependent Protein Kinase (DNA-PK) in Ageritin-Treated Glioblastoma Cells

**Figure 6:**
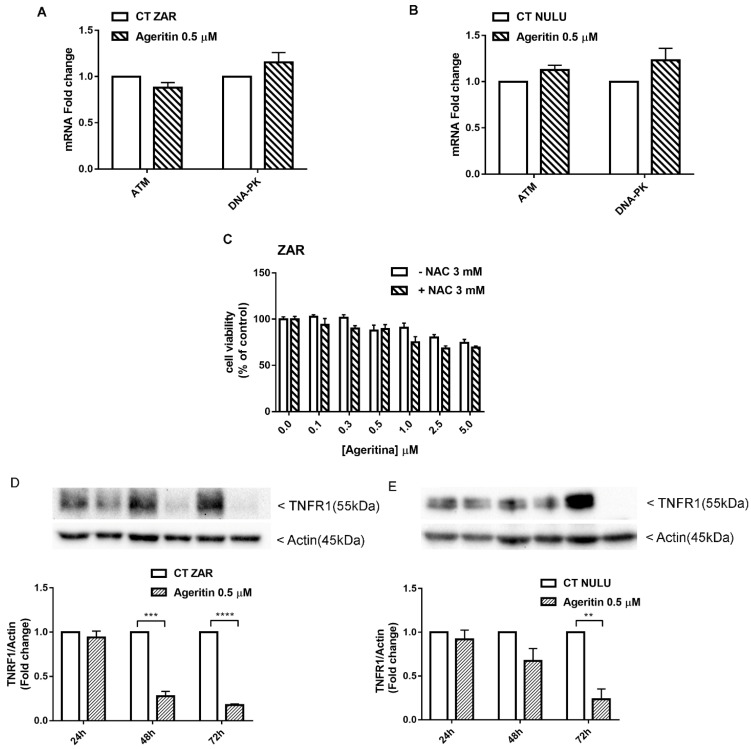
Potential mechanisms involved in Ageritin-induced cytotoxicity in patient-derived glioblastoma cells NULU and ZAR. (**A**,**B**) Quantitative RT-PCR of ATM and DNA-PK in NULU and ZAR cells treated with Ageritin 0.5 μM for 48 h. Untreated cells were used as the control. Data did not reveal any statistically significant change in the analyzed target genes. (**C**) Pretreatment of ZAR cell line with NAC 3 mM for 4 h followed by Ageritin 0.1, 0.25, 0.5, 1, 2.5 and 5 μM. Untreated cell were used as control. (**D**,**E**) Western blot analysis of TNFR1 protein of long-term-treated ZAR and NULU cells with Ageritin 0.5 μM for 24, 48 and 72 h. Normalization was performed with housekeeping gene Actin. Densitometric analysis of protein levels represent the means ± SEM of 3 individual determinations. Data are expressed as fold change over control-treated cells. Unpaired *t*-test, *p*-value < 0.05. According to GraphPad Prism 7, ** *p*-value 0.001 to 0.01 (very significant), *** *p*-value 0.0001 to 0.001 extremely significant, **** *p*-value < 0.0001 extremely significant.

The double-strand break (DSB) response pathway involves, among others, two members of the family of phosphatidyl inositol 3-kinase-like kinases (PIKKs), which are ATM and DNA-PK, both relevant in DSB repair, induced by ionized radiation (IR) and chemotherapy [22,23]. In this regard, the ability of Ageritin to deregulate the expression of ATM and DNA-PK was evaluated on both ZAR and NULU cell lines. As shown in Figure 6A,B, quantitative RT-PCR did not reveal any change in ATM and DNA-PK transcript levels in ZAR and NULU patient-derived glioblastoma cell lines treated with 0.5 µM Ageritin for 48 h.

### 2.6. Potential Role of Ageritin in Oxidative Stress and Deregulation of Tumour Necrosis Factor Receptor 1 (TNFR1)

The MN-γ-H2AX (+) increase in patient-derived glioblastoma cells treated with Ageritin, led us to suppose the involvement of reactive oxygen species as a potential cause of DNA lesions [24]. Since the antioxidant N-acetylcysteine (NAC) has been reported to attenuate the cellular frequency of MN-γ-H2AX (+), Ageritin-induced cytotoxicity by oxidative stress was evaluated by preincubating the most responsive cell line ZAR with NAC. As shown in Figure 6C, the antioxidant NAC is not able to revert the effect of Ageritin, leading us to exclude the involvement of oxidative stress as an Ageritin-mediated mechanism. The exclusion of oxidative stress was supported by the surprising observation of a complete ablation of TNFR1 expression after 72 h of Ageritin treatment in ZAR and NULU cell lines, as reported in Figure 6D,E. However, the blockage of TNFR1 expression did not affect the downstream signaling of NF-κB, since neither the protein expression of p65 nor p50 decreased under Ageritin treatment (Figure S1).

### 2.7. The inhibition of MGMT Protein Expression and Sensitization of Primary Glioblastoma Cells Lines to TMZ after Ageritin Pre-Treatment

**Figure 7:**
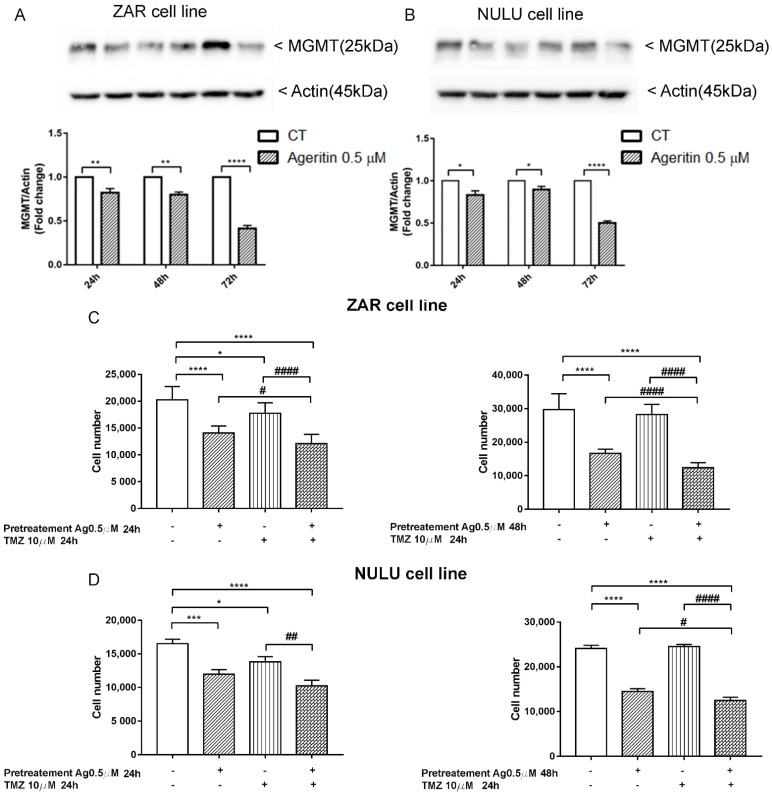
Combinatorial effect of Ageritin and TMZ on ZAR and NULU glioblastoma cell lines. (**A**,**B**) Western blot analysis of MGMT protein of long-term treated ZAR and NULU cells with Ageritin 0.5 µM for 24, 48 and 72 h. Normalisation was performed with housekeeping gene Actin. Densitometric analysis of protein levels represent the means ± SEM of 3 individual determinations. Data are expressed as fold change over control-treated cells. (**C**,**D**) Pretreatment of NULU and ZAR cell lines with Ageritin 0.5 µM followed by TMZ 10 µM for 24 h. Cells treated with TMZ 10 µM for 24 h and Ageritin 0.5 µM for 24 and 48 h as single agents, and untreated cells were used as the control. Data are reported as mean ± SEM of 3 individual determinations. Unpaired *t*-test, *p*-value < 0.05. According to GraphPad Prism 7, * or # *p*-value 0.01 to 0.05 Significant, ** or ## *p*-value 0.001 to 0.01 (very significant), *** *p*-value 0.0001 to 0.001 extremely significant, **** or #### *p*-value < 0.0001 extremely significant.

Previous molecular characterization of NULU and ZAR patient-derived glioblastoma cell lines revealed the unmethylated profile of the MGMT gene promoter [18] and, consequently, a pharmaco-resistance phenotype to standard chemotherapy with TMZ. Therefore, in the light of possible future clinical applications, we investigated the expression of MGMT protein under Ageritin exposure. Western blot analysis of long-term treated glioblastoma cells revealed the decrease of MGMT protein for both ZAR and NULU cell lines (Figure 7A,B, respectively). Therefore, considering both the increased frequency of MN-γ-H2AX (+) reported above (Section 2.4) and the MGMT expression reduction under the same conditions, we decided to evaluate the responsiveness of ZAR and NULU cell lines to TMZ, after pre-treatment with 0.5 µM Ageritin for 24 and 48 h. Cell counting revealed a promising increase in the sensitivity to TMZ of both patient-derived glioblastoma cell lines, and sensitivity after 48 h of Ageritin pre-treatment (Figure 7C,D).

## 3. Discussion

In a previous work, the neurotoxin effect of Ageritin towards either undifferentiated or retinoic acid (RA)-differentiated SH-SY5Y neuroblastoma cells showing a selective toxicity against undifferentiated cells was reported [14]. In light of this, considering the possibility of using Ageritin as a specific neurotoxin, we tested its toxicity on ZAR and NULU primary human glioblastoma cells derived from the mechanical and enzymatic digestion of patient biopsies. According to WHO CNS 2016 classification, NULU and ZAR are IDH1 wild-type glioblastoma cell lines with unmethylated MGMT promoter genes, leading to a drug-resistant phenotype [18]. ZAR and NULU cell lines treated with Ageritin (0.25 and 0.5 μM) for 24, 48 and 72 h resulted in a significant inhibition of growth, as shown by the daily cell count (Figure 2B,E). Moreover, growth curves highlighted a higher response of the ZAR cell line, which significantly decreased after 24 h of Ageritin treatment—as also confirmed by MTT toxicity assay (Figure 2A,D). This different response of ZAR and NULU cell lines to Ageritin treatment is attributable to the intrinsic diversity of the two primary human glioblastoma cell lines, which reproduced the intrinsic physiological diversity of glioblastoma in vitro [18]. Moreover, Ageritin significantly inhibited ZAR and NULU cell migration in the wound healing assay (Figure 3A,B). Surprisingly, the Ageritin treatment of patient-derived glioblastoma cell lines lead to a macroscopic genotoxic response through MN formation, which represented the adaptive response to the genomic chaos of cells exposed to a genotoxic agent [25], as occurred for Ageritin (Figure 4). Specifically, the treatment of both ZAR and NULU cell lines with Ageritin for 48 h showed a significant increase in MN formation of about 40%, with respect to the untreated cells used as the control. The presence of MN, detected by DAPI counterstaining, was consolidated by the increasing detection of γ-H2AX, a marker of DNA double-strand breaks (Figure 4A), accordingly to that previously reported in [26]. A further test with Cytochalasin B confirmed our hypothesis that Ageritin can cause direct cancer cell DNA damage (Figure 5).

Increased MN frequency induced by Ageritin reflects the genotoxic behavior of different anti-cancer drugs, such as Adriamycin [27,28], gemcitabine and topotecan [29], and bleomycin, whose effect was enhanced by the DNA-PK inhibitors wortmannin [30] and vindesine before exposure to gamma-radiation [31]. In our study, despite the presence of MN-γ-H2AX (+), DNA damage was not supported by either the deregulation of DDR enzymes ATM and DNA-PK or oxidative stress induction, since glioblastoma cells treated with antioxidant NAC did not revert the Ageritin-induced cytotoxicity (Figure 6). The involvement of reactive oxygen species (ROS) in MN-γ-H2AX (+) increasing with the Ageritin-mediated mechanism was further excluded by the complete ablation of TNFR1 (Figure 6D,E). Indeed, it is well-known that ROS are important regulators of TNF-TNFR signaling, and the binding of soluble TNF to TNFR1 can lead to the activation of factor NF-κB transcription, driving different signaling [32]. However, the blockage of TNFR1 expression did not affect the downstream pathway involving p65 and p50 NF-κB subunits. Considering the genotoxic effect of Ageritin, we verified the sensitivity to TMZ of both patient-derived glioblastoma cell lines after Ageritin pre-treatment (Figure 7C,D). In addition, as demonstrated by the Western blot analysis, a decrease in MGMT protein for both NULU and ZAR cell lines treated with Ageritin was evident (Figure 7A,B, respectively), further explaining why Ageritin pre-treatment increased the inhibition of NULU and ZAR growth mediated by the treatment with TMZ. The inhibition of MGMT in glioblastoma cells treated with Ageritin showed a direct correlation between Ageritin treatment and DNA damage and the different inhibition times between the two cell lines NULU and ZAR, highlighting the heterogeneity of the response of the glioblastoma to chemotherapy. Moreover, the partial resistance to proteolysis at a neutral pH, using an in vitro digestion system verified in this work, represents an important physicochemical characteristic of Ageritin. This novel finding, combined with the previously proved high thermal and chemical stability of Ageritin [6,7], promotes this RL-P as a rising candidate for the synthesis of bio-conjugates, obtained using carrier antibodies or other specific molecules (e.g., peptides, hormones or nanoparticles). It is well known that that the cytotoxicity of protein toxins is correlated to their intrinsic resistance to endogenous endoproteinases, which often cause inactivation over a period of action [33,34].

It is very difficult to establish the specific mechanism of Ageritin in inhibiting the growth of glioblastoma cells. As ribotoxin-like proteins (RL-Ps), Ageritin commonly works by cleaving a single phosphodiester bond located within the universally conserved alpha-Sarcin Ricin Loop (SRL) of 23-28S rRNAs. This cleavage leads to the inhibition of protein biosynthesis: in our study, we found the total inhibition of TNFR1 and, to a minor extent, the inhibition of DNA repair protein MGMT. Even though Ageritin did not mediate the inhibition of DNA damage repair enzymes ATM and DNA-PK, it could not exclude the blockage of other DNA repair mechanisms, leading to genotoxic effects, evaluable as the increased frequency of MN-γ-H2AX (+). Therefore, we considered these features very interesting, and propose Ageritin as an adjuvant substance for the treatment of glioblastoma on several fronts: on the one hand, the genotoxicity of Ageritin is similar to many anti-cancer drugs; on the other hand, it inhibits TNFR1, which directs the control of the life and death balance in a cell. Moreover, the inhibition of MGMT, which is mainly responsible for resistance to chemotherapy drugs, contributes to ameliorating the TMZ response.

Overall, both biological and physicochemical findings reported in this work confirm the possible usefulness of Ageritin as a cytotoxic tool for the construction of immunotoxins/conjugates designed for a possible targeted therapy against GBM. Indeed, in recent years, immunotoxins/conjugates have been developed using protein toxins and a variety of carriers are endowed with specificity for different targets, verifying the concept of “magic bullets” formulated by Paul Ehrlich [35].

## 4. Materials and Methods

### 4.1. Materials

Materials for chromatography have been described elsewhere [3,36]. All other reagents and chemicals (e.g., MTT assay (3-(4,5-dimethylthiazol-2-yl)-2,5-diphenyltetrazolium); isopropanol; N-acetyl-cysteine; 0.4% trypan blue solution; tween-20; Triton X-100) were of analytical grade (Sigma-Aldrich/Merck Life Science S.r.l., Milano, Italy), and 10% Neutral Buffered Formalin was purchased from Diapath (Diapath, Martinengo, Italy). All reagents for cell culture (e.g., DMEM, FBS, streptomycin/penicillin) were from EuroClone (Milan, Italy).

### 4.2. Purification of Ageritin

Ageritin was purified according to the procedure previously reported [3]. Briefly, the raw extract of *C. aegerita* fruiting bodies was acidified with acetic acid and subjected to consecutive chromatographic steps: Streamline-SP (GE Healthcare, Milano, Italy) step wise; gel-filtration by Sephadex G-75 Hi-load 26/60 column (GE Healthcare) on an Akta purification system. Finally, a low-pressure cation exchange chromatography step on an SP-Sepharose (GE Healthcare) was eluted with an increasing linear NaCl gradient. Fractions corresponding to the principal main peak (Ageritin) able to release the α-fragment without aniline treatment when incubated with rabbit ribosomes were checked by SDS-PAGE analysis, pooled and dialyzed against water, freeze-dried, and stored at −20 °C until use.

### 4.3. In Vitro Proteolytic Digestion

Samples for proteolytic digestion were prepared by mixing 200 µL of buffer with 60 µg of Ageritin in the presence of the proteolytic enzyme with a protease stoichiometric ratio of 5:1, based on previous observations/experiments [13,19]. Proteases and buffers used were: pepsin in 20 mM sodium citrate, pH 3.0; trypsin or chymotrypsin in 50 mM ammonium bicarbonate, pH 8.0. The reactions were performed for 10, 30 and 60 min at 37 °C and were stopped by thermal denaturation (10 min at ~100 °C). Subsequently, the samples were mixed with an equal volume of denatured loading buffer for SDS-PAGE, and suitable aliquots (~10 µg of Ageritin) were subjected to denatured SDS-PAGE (15% polyacrylamide).

### 4.4. Cell Cultures

Human patient-derived glioblastoma cell lines were established from bioptic samples from patients who gave informed consent to participate in the study. The use of primary cell lines as a model for GBM heterogeneity was approved by the Ethics Committee on 27 February 2020 and registered on ClinicalTrials.gov with the identification number NCT04180046. Glioblastoma cell lines NULU and ZAR, used in the following experiments, were characterized as previously reported [18,37]. In detail, primary glioblastoma cells NULU and ZAR were cultured in Dulbecco’s Modified Eagle’s Medium (DMEM) supplemented with 10% fetal bovine serum (FBS), 2 mmol/L-glutamine, 100 IU/mL penicillin, 100 μg streptomycin at 37 °C, 5% CO_2_, and 95% humidity.

### 4.5. Cytotoxicity Test and IC_50_ Values

In order to evaluate the cytotoxic effects of Ageritin, patient-derived glioblastoma cells NULU and ZAR were plated at a density of 5 × 10^3^ cells/well in 96-well plates. Cells were treated with Ageritin at 0.001, 0.01, 0.1, 1, 2.5 and 5 µM for 48 h and IC_50_ values were estimated with GraphPad Prism 7 software (GraphPad Software Inc, La Jolla, CA, USA). According to IC_50_ values, cells were daily treated with Ageritin 0.25 and 0.5 µM for 24, 48, and 72 h. The MTT assay was performed by adding 5 mg/mL of formazan salts to 100 µL of cells cultured in DMEM with FBS 10%. The formazan crystals were dissolved with 0.4% isopropanol/HCl and the absorbance was measured at 595 nm with a plate reading spectrophotometer.

### 4.6. Growth Curve and Cell Proliferation Assay by Trypan Blue

Growth rate of patient-derived glioblastoma cells NULU and ZAR treated with Ageritin were plated in 48-well plates at 1 × 10^4^ cells/well in DMEM supplemented with 10% FBS and incubated at 37 °C in an atmosphere containing 5% CO_2_. Ageritin 0.25 and 0.5 µM was administrated daily to primary glioblastoma cells and counts were performed at 24, 48 and 72 h of treatment. Viable cell counting was performed by the trypan blue exclusion method at the higher concentration of ribotoxin-like protein used (Ageritin 0.5 µM) at 24, 48 and 72 h of treatment. Cell viability (%) was calculated as follows:

(1) $$\mathrm{Cell}\;\mathrm{viability}\;\left(\%\right)=\frac{\mathrm{total}\;\mathrm{viable}\;\mathrm{cells}\;(\mathrm{unstained})}{\mathrm{total}\;\mathrm{cells}\;(\mathrm{stained}\;\mathrm{and}\;\mathrm{unstained})}\times \;100$$

### 4.7. Wound Healing Assay

Primary glioblastoma cell lines NULU and ZAR were plated in 6-well plates at a density of 2.5 × 10^5^ cells/well in DMEM with 0.5% FBS for 48 h. The scratch assay was performed as previously reported [38]. Briefly, a linear thin scratch “wound” was performed in a confluent cell monolayer of primary cell lines and detached cells were removed by washing with PBS. Cell motility, untreated or treated with 0.5 µM Ageritin, was monitored and imaged under a EVOS FL microscope (Life Technologies) for each time point (T0, T6, T24, T48, T72 and T96 h). Scratch area was quantified by ImageJ software v. 1.53 and reported as wound closure (%).

### 4.8. Immunofluorescence for Micronuclei (MN)-γ-H2AX (+)

The evaluation of MN-γ-H2AX (+) was performed by the immunofluorescence staining of patient-derived glioblastoma cells NULU and ZAR. In detail, 1 × 104 cells were seeded in 8-well chamber slides in DMEM with 0.5% FBS for 48 h. Media was then replaced with DMEM with FBS 10% and cells were treated with 0.5 µM Ageritin for 48 h. At the end of treatment, cells were washed twice with phosphate-buffered saline (PBS) and fixed in 4% formalin for 20 min and permeabilized with 0.1% Triton for 30 min. After blocking with 10% specific serum, the cells were incubated with antibody against phospho-H2AX (Ser139), or γ-H2AX, (Cell signaling, 1:400) overnight at 4 °C. After washing with 0.025% PBS-Tween-20, cells were incubated with secondary antibody anti-mouse fluorescein (1:100; Vector, Stuttgart, Germany) in 2% serum for 1 h at room temperature. The slides were counterstained with DAPI Mounting Medium (Vectashield) for nuclei and micronuclei detection with an EVOS FL microscope. Nuclei were scored first for MN by their DAPI staining under a 60× magnification, and then for the presence or absence of γ-H2AX signals. Data were reported as mean ± SEM of number of MN-γ-H2AX (+) per 100 cells.

### 4.9. Cytochalasin B Micronucleus Assay

The evaluation of MN-γ-H2AX (+) in binucleated cells was performed seeding 1 × 10^4^ cells in 8-wells chamber slides in DMEM with 0.5% FBS for 48 h. Cells were pretreated with 3 nM Cytochalasin B for 16 h; media was then replaced with Cytochalasin-free DMEM with FBS 10% and cells treated with 0.5 µM Ageritin for 48 h. Control cells (untreated with Ageritin) were also incubated with Cytochalasin B. Immunofluorescence staining and MN-γ-H2AX (+) detection in binucleated cells was performed as described in Section 4.8. Data were reported as mean ± SEM of number of MN-γ-H2AX (+) per 100 binucleated cells.

### 4.10. Real-Time PCR for DNA Damage Repair Enzymes ATM and DNA-PK

Total RNA was isolated using TRIzol^TM^ Reagent (Thermo Fisher Scientific, Waltham, MA, USA) followed by Direct-zol^TM^ MiniPrep (Zymo Research, Irvine, CA, USA) according to the manufacturer’s instruction. Potential DNA contamination was removed by RNase-free DNase treatment. cDNA was obtained by reverse transcription-PCR of 1 µg of total RNA using the SuperScript III First-Strand Synthesis System for RT-PCR (Invitrogen). Real-time RT-PCR was performed using the SYBR green PCR Master Mix (Applied Biosystems) in the CFX Connect Real-Time System (Bio-Rad). Human GAPDH gene was used as the control. Primer sequences for ATM and DNA-PK and GAPDH are described below:
**Target****Forward Primer****Reverse Primer**ATM5′-TTTACCTAACTGTGAGCTGTCTCCAT-3′5′-ACTTCCGTAAGGCATCGTAACAC-3′DNA-PK5′-CCAGCTCTCACGCTCTGATATG-3′5′-CAAACGCATGCCCAAAGTC-3′GAPDH5′-GGTGAAGGTCGGAGTCAA-3′5′-CATGTAGTTGAGGTCAATGAA-3′

### 4.11. Western Blot Analysis

Patient-derived glioblastoma cells were plated at a density of 5 × 10^5^ cells in 60 mm plates in DMEM (with FBS 0.5%) for 48 h. Media were replaced with fresh DMEM with 10% FBS and Ageritin 0.5 µM administrated daily. Cells were collected at 24, 48 and 72 h of treatment. Change in the protein expression profile was assessed by the Western blot analysis of protein extract obtained by the lysis of the cells with Triton X-100 lysis buffer (10 mM Tris-HCl, 1.0 mM EDTA, 150 mM NaCl, 1% Triton X-100, NaF 1.0 mM, 1.0 mM Na_4_P_2_O_7_, 1.0 mM Na_3_VO_4_ and 1× protease inhibitors). Protein concentration was determined with the Bradford assay, and samples (15 µg) were separated by SDS-PAGE and transferred to PVDF membranes by electroblotting. The membranes were blocked for 1 h at room temperature with 5% non-fat dry milk or BSA (bovine serum albumin) diluted in Tris 1× buffered saline containing Tween-20 (TBST), and subsequently incubated with specific primary antibodies overnight at 4 °C. Mouse monoclonal anti-β-actin (1: 10,000, Santa Cruz Biotechnology, Dallas, TX, USA) was used for protein normalization, incubating the membrane for 1 h at room temperature. The membranes were then exposed to secondary antibodies conjugated with the HRP enzyme (Calbiochem, Merk Life Science Srl, Milan, Italy). The protein bands were visualized by the chemiluminescence using ECL Western blotting (GE healthcare Life Sciences, Milan, Italy), while the digital signals were quantified by densitometric analysis using the Image Lab software 6.1 for Windows (Bio-Rad Laboratories, Rome, Italy). The membranes were incubated with the antibodies anti-MGMT (1:500, Cell Signaling Technology, Danvers, MA, USA), anti-TNFR1 (1:1000, Cell Signaling Technology), anti-NF-κB p65 subunit (1:1000, Santa Cruz, CA, USA) and anti-NF-κB p50 subunit (1:1000, Santa Cruz, CA, USA).

### 4.12. Involvement of Oxidative Stress in Ageritin-Mediated Signals

Ageritin-induced oxidative stress was assessed by plating primary glioblastoma cell line ZAR, the most sensitive to Ageritin, at a density of 5 × 10^3^ cells/well and starved for 48 h in DMEM with 0.5% FBS. After pre-treatment with the antioxidant N-acetylcysteine (NAC) 3 mM for 4 h at 37°C in DMEM with 10% FBS, the medium was replaced with fresh DMEM and cells were treated with Ageritin at concentrations of 0.1, 0.25, 0.5, 1, 2.5 and 5 µM, using untreated cells as the control. The effect of NAC to reverse Ageritin treatment was determined by MTT assay, as described in Section 4.5.

### 4.13. Sensitivity Response of Patient-Derived Glioblastoma Cells to TMZ, after Pre-Treatment with Ageritin

The effects of Ageritin to sensitize the in vitro response of primary glioblastoma cells NULU and ZAR to TMZ were evaluated by seeding cells in 48-well plates (1 × 10^4^ cells/well) in DMEM with FBS 0.5% for 48 h. Media were replaced with DMEM supplemented with FBS 10% and cells were pre-treated with 0.5 µM Ageritin for 24 and 48 h followed by 24 h of exposure to TMZ 10 µM. Untreated cells were used as the control, and cells were treated with 0.5 µM Ageritin and/or TMZ 10 μM for 24 and 48 h. At the end of each treatment, cells were counted using a Burker chamber to detect cell number after treatment.

### 4.14. Statistical Analysis

Experiments were performed in triplicate, data were expressed as mean ± SEM and were analyzed by Student’s *t*-test or one-way ANOVA. The differences were considered significant if *p* < 0.05. Analyses were carried out using the GraphPad Prism 7 software (GraphPad Software Inc.).

## 5. Conclusions

This study opens to a novel approach for GMB adjuvant therapy. Interestingly, Ageritin is a natural Ribotoxin-like protein that revealed to control GBM growth in vitro, especially in cells with unmethylated MGMT promoter, that exhibit a pharmaco-resistance phenotype. 

## Data Availability

The raw data supporting the conclusions of this article will be made available by the authors upon request, without undue reservation.

## Supplementary Materials

The following supporting information can be downloaded at: https://www.mdpi.com/article/10.3390/molecules27082385/s1, Figure S1: Western blot analysis of NF-κB p50 and p65 subunits in long-term Ageritin-treated patient-derived glioblastoma cell lines ZAR and NULU.

## Sample Availability

Ageritin enzyme is available on request from antimo.dimaro@unicampania.it, NULU and ZAR cell lines information is available from arcella@neuromed.it (Neuromed).
