# Supplementary material for: Ageritin—The Ribotoxin-like Protein from Poplar Mushroom (Cyclocybe aegerita) Sensitizes Primary Glioblastoma Cells to Conventional Temozolomide Chemotherapy
Source: Molecules. 2022 Apr 7;27(8):2385. doi: 10.3390/molecules27082385 (PMC9032345; doi:10.3390/molecules27082385)
Supplement: Supplementary file 1 [file molecules-27-02385-s001.zip › molecules-1650794-supplementary.pdf]

molecules

Article

Ageritin—The Ribotoxin-like Protein from Poplar Mushroom (*Cyclocybe aegerita*) Sensitizes Primary Glioblastoma Cells to Conventional Temozolomide Chemotherapy

Rossella Rotondo [1,†], Sara Ragucci [2,†], Salvatore Castaldo [1], Nicola Landi [2], Maria Antonietta Oliva [1], Paolo V. Pedone [2], Antimo Di Maro [2,*,‡] and Antonietta Arcella [1,*,‡]

[1] IRCCS Istituto Neurologico Mediterraneo NEUROMED, Via Atinense 18, 86077-Pozzilli, IS, Italy; rossellaross1988@gmail.com (R.R.); castaldosal90@gmail.com (S.C.); mariaantonietta.oliva@neuromed.it (M.A.O.)
[2] Department of Environmental, Biological and Pharmaceutical Sciences and Technologies (DiSTABiF), University of Campania "Luigi Vanvitelli", Via Vivaldi 43, 81100-Caserta, Italy; sara.ragucci@unicampania.it (S.R.); nicola.landi@unicampania.it (N.L.); paolovincenzo.pedone@unicampania.it (P.V.P.)
* Correspondence: antimo.dimaro@unicampania.it (A.D.M.); arcella@neuromed.it (A.A.)

† These authors contributed equally to this work.
‡ These authors share equal senior authorship.

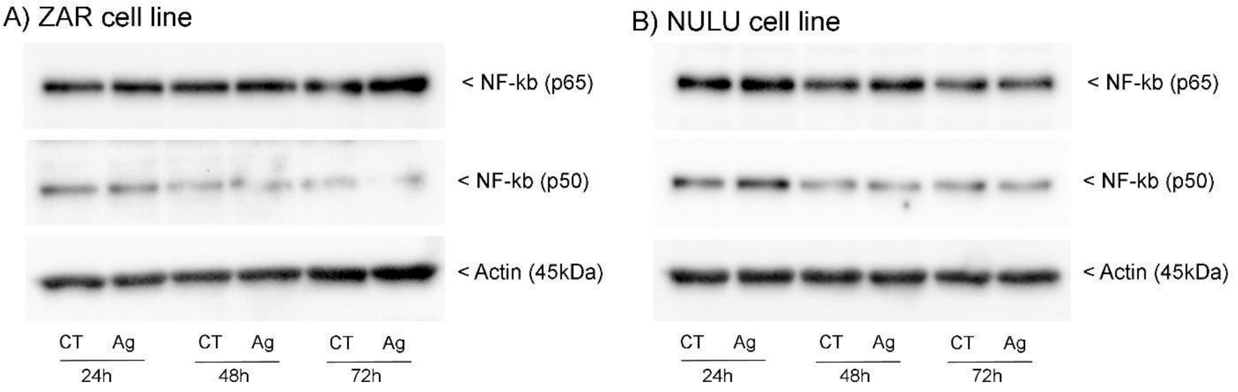

Figure S1: Western blot analysis of NF-κB p50 and p65 subunits in long-term Ageritin-treated patient-derived glioblastoma cell lines。
